# Prevalence of musculoskeletal disorders among police officers from an organizational unit of a German federal state police force

**DOI:** 10.1186/s12995-026-00511-x

**Published:** 2026-04-17

**Authors:** Janna Schlenke, Yunes Nazzal, Faiz Dogru, Fabian Holzgreve, Rejane Golbach, Ioannis Karrasavidis, Ulli Brand, Eileen M. Wanke, David A. Groneberg, Daniela Ohlendorf

**Affiliations:** 1 Institute of Occupational Medicine, Social Medicine and Environmental Medicine, Goethe-University Frankfurt am Main, Theodor-Stern-Kai 7, Building 9A, 60596 Frankfurt am Main, Germany; 2 Institute of Biostatistics and Mathematical Modelling, Goethe-University, Theodor-Stern-Kai 7, 60596 Frankfurt am Main, Germany; 3 Health Care Management, Organizational Unit Federal State Police, Berlin, Germany; 4 Organizational unit federal state police, Berlin, Germany

**Keywords:** Musculoskeletal disorders, Police officers, Germany, Nordic musculoskeletal questionnaire

## Abstract

**Background:**

Occupational activities involving prolonged postures, restricted movements, and wearing heavy protective equipment contribute significantly to work-related musculoskeletal disorders (MSDs) in police officers. Therefore, this study aims to assess the prevalence of MSDs at an organizational unit of a German federal state police force in relation to self-reported causes of MSD.

**Methods:**

255 (211 m/44 f) participants within the age from 21 to 57 years (28.29 ± 6.57 years) answered a modified version of the Nordic Musculoskeletal Questionnaire (NMQ) in nine different body areas (cervical spine/neck, thoracic spine, lower back/lumbar spine, shoulder, elbow, hand and wrists, hip, knee, foot and ankles). For statistical analyses descriptive analyses, Spearman’s rank correlation coefficient and the Rasch model analyses were applied. Significance level was set at 5%.

**Results:**

Of the 255 participants 95.7% engage in physical activity during their leisure time, with the vast majority (78.1%) also involved in occupational sports activities. The overall 12-month prevalence of MSD was 92.9%, while the most affected areas were the lower back (64%), followed by the neck/cervical spine (57.8%), shoulder (48.6%) and the thoracic spine (33%). The 7-day prevalence stands at 56.1%. The regions most commonly affected are similar to the 12-month prevalence: the neck/cervical spine with 25.1%, followed by the lower back (20.4%), the shoulder (13.7%), and the thoracic spine (11.8%). Only 7.1% reported no symptoms at all. 1.6% indicated experiencing discomfort in all body regions. The Rasch-derived musculoskeletal symptom score showed significant positive correlations with age (*r* = 0.17, *p* < 0.01), BMI (*p* < 0.01, rho = 0.19), and years of service (*r* = 0.20, *p* < 0.01). Participants most frequently attributed their musculoskeletal complaints to carrying heavy equipment, prolonged sitting, and awkward postures, followed by stress and insufficient recovery. Only a minority associated their symptoms with sports or physical training, suggesting that ergonomic and organizational factors are perceived as the primary contributors to MSDs.

**Conclusion:**

Musculoskeletal disorders are highly prevalent among German police officers, affecting nearly all participants despite a young mean age and high physical activity levels. The predominance of multi-site pain, particularly in the lower back, neck, and shoulders, highlights the cumulative impact of occupational load rather than isolated risk factors. Equipment-related load carriage, prolonged static postures, and organizational demands appear to be key drivers, while age, BMI, and years of service exert additional but modest influence. These findings underscore the need for early, occupation-specific preventive strategies that integrate ergonomic optimization, task organization, and targeted physical training to preserve work ability and operational readiness over the course of a policing career.

## Background

Musculoskeletal disorders (MSDs) constitute a major health problem for the working population and represent a substantial economic burden in occupational settings [1]. They are among the leading causes of reduced work ability and productivity across industries [1–7]. In Germany, production loss costs amounted to 2.9% of gross national income in 2022, with MSDs accounting for approximately 0.5% of these losses and 18.2% of all sick-leave diagnoses, highlighting considerable potential for preventive interventions [1]. Stressful physical and psychosocial work characteristics often serve as precursors to MSDs [3, 4, 8, 9]. Conversely, physical pain may also function as a mediator between overcommitment, effort–reward imbalance, and depression [10].

Certain professions are at particularly high risk for MSDs due to their working conditions, such as police officers. Their occupation is physically demanding and involves physical confrontation, wearing heavy protective gear, prolonged sitting in vehicles, and other static and repetitive positions [2, 11–14]. Overall, the demands on German police officers have increased in recent years, with a corresponding rise in musculoskeletal complaints [15–17].

Marins et al. [12] showed that international evidence consistently demonstrates a high prevalence of MSDs among police officers, typically ranging between approximately 40% and 70%, depending on the body region, reference period, and population studied. Across countries, the lumbar spine emerges as the most frequently affected region (approximately 41–68%), followed by the cervical spine (29–57%), thoracic spine (22–45%), knees (32–52%), and shoulders (7–32%) [12, 13, 18–21]. These prevalence estimates have predominantly been derived from standardized self-report instruments, most commonly the Nordic Musculoskeletal Questionnaire (NMQ) [22] or validated national adaptations thereof [20, 21, 23], as well as large occupational health surveys such as the Swedish Work Environment Survey (SWES) [2, 8].

Country-specific studies further substantiate this overall pattern. Elevated symptom prevalences have been reported among police officers in Brazil [18], Sweden [8, 19], Poland [20], Canada [13, 21], and the United States [24], frequently linked to occupation-specific demands such as prolonged vehicle use, motorcycle patrols, or mandatory equipment. While absolute prevalence estimates vary across policing contexts and subunits, the reported values consistently fall within the aforementioned international ranges [2, 12–21].

Within Germany, available evidence indicated a comparable MSD burden among police officers [9, 25]. Studies of special police units (German abbreviation: SEK, MEK) reported 12-month prevalence rates of approximately 42% for neck pain, 34% for shoulder pain, and over 50% for back pain, assessed using an adapted non-standardized population-based survey focusing on four body regions (neck, shoulder, back and hip) [3]. Health monitoring data from Berlin police officers similarly showed 12-month prevalence rates of around 41% for back pain and 43.5% for neck/shoulder pain, measured using the German SALSA questionnaire supplemented with police-specific items [15].

Beyond occupational exposures, individual characteristics such as age and body mass index (BMI) are potentially determinants of musculoskeletal disorders (MSDs) in both the general and working populations, and contribute to the global burden of MSD-related disability [26]. Increasing age has consistently been associated with higher MSD prevalence and severity, which has been attributed to cumulative biomechanical strain, age-related degenerative changes, and a reduced capacity for tissue repair and recovery [27].

Elevated BMI further exacerbate MSD risk through increased mechanical loading of the musculoskeletal system and systemic low-grade inflammation. Evidence from a large Dutch cohort study with the working population demonstrated that overweight and obesity were associated with higher 12-month MSD prevalence, increased risk of symptom onset, and reduced likelihood of recovery [28]. These findings are supported by meta-analytic evidence showing that increasing BMI elevates MSD risk across multiple body regions, including non-weight-bearing joints, indicating that metabolic and inflammatory mechanisms play a role in addition to mechanical overload [29]. At the general population level, high BMI has been estimated to account for approximately 10.3% of MSD-related disability-adjusted life years worldwide, underscoring its relevance as a modifiable risk factor [26].

Despite existing national research, significant gaps remain. Existing studies among German police officers [3, 15] vary considerably with respect to the police subpopulations examined, assessment instruments used, and the extent to which individual and occupational risk factors are jointly considered. Moreover, the added value of combining standardized MSD assessment tools with police-specific items addressing operational demands and health-related consequences has not been sufficiently examined.

The NMQ has proven to be a valid and widely used instrument for assessing musculoskeletal complaints across occupational groups, including police officers [2, 8, 22], and was thus chosen and adapted for this study. The aspect of how psychosocial factors contribute to MSDs is included in a larger research project conducted by our group, which will also include the investigation of postural patterns and their association with MSDs [30, 31].

Accordingly, the present study aims to determine the prevalence of MSDs among an organizational unit of a German federal state police force, and assesses associations with age, BMI, and years of service. Therefore, the hypotheses to be tested are:

### H1

Increasing age will be associated with higher MSD severity.

### H2

A higher BMI will be associated with increased MSD severity.

### H3

A greater number of working years in active police service will be positively associated with more severe MSDs.

## Materials and methods

### Subjects

255 (211 m/44 f) police officers (mean age 28.3 ± 6.6 years) from specialized tactical units of a single federal state police force in Germany voluntarily participated in the study. Those men and women included in this study were between 18 and 65 years old and engaged in active service. The demographic and gender specific characteristics are shown in Table 1. Almost all (*n* = 244) engage in at least 3–4 h of sports per week in their leisure time, primarily weight training/fitness, martial arts, soccer, and various forms of endurance sports. In contrast, 78.1% (*n* = 200) participate additionally in sports as part of their job (on average 1–3 h running and strength training). On average, participants have been serving in their respective occupations for 5.2 ± 6.5 years, committing approximately 43 h per week to work.

**Table 1:** Demographic characteristics of the study sample of all subjects and when separated by sex

	All subjects (*n* = 255)				Male (*n* = 211)				Female (*n* = 44)			
	Mean value	SD	Median	1st/3rd Quartil	Mean value	SD	Median	1st/3rd Quartil	Mean value	SD	Median	1st/3rd Quartil
Age, years	28.3	6.6	26.0	24.0/31.0	29.1	6.9	27.0	24.0/32.0	24.5	2.3	24.0	23.0/25.5
Body height, cm	180.2	8.3	180.0	175.0/185.0	182.6	6.6	182.0	178.0/187.0	166.3	14.70	168.0	164.8/172.0
Body weight, kg	81.9	12.4	83.0	74.0/89.5	87.6	30.1	85.0	80.0/91.0	63.91	5.85	63.0	59.0/68.5
BMI, kg/m²	26.1	2.37	25.0	23.4/26.7	26.3	7.3	25.6	24.2/27.2	22.3	1.7	24.0	21.4/23.7

Given the diverse nature of police work across departments, the study specifically focused on officers with at least some level of physical capability to cope the demanding work conditions. This included scenarios such as wearing up to 20 kg of body protection gear during duty or routinely encountering physical confrontations.

Consequently, individuals were included in the study only if they exhibited unimpaired physical performance on the day of measurement. Pre-existing musculoskeletal conditions, known to the participants and not limiting their ongoing job performance within the studied units, did not serve as exclusion criteria. However, those officers experiencing temporary or permanent performance impairments preventing participation in active duty, as certified by a work incapacity certificate, were excluded.

In advance, the department heads were informed about the forthcoming study and its general procedure through an on-site briefing. The purpose of the briefing was to ensure adherence to official organizational structures and processes, since the police department provided time during working hours for the study. The participants gained access to the questionnaire through emails by the internal police force health department, and were able to register at the same time.

All participants signed an informed consent form. A positive ethics vote has been obtained from the Department of Psychology and Sports Science at Goethe University Frankfurt am Main n accordance with the Declaration of Helsinki for the conduct of the study (File number: 2022-07).

### Questionnaire

The online questionnaire was made available through the SoSci-Survey portal, ensuring accessibility for all participants. A declaration of informed consent had to be accepted online at the beginning of the survey. Using the SoSci Survey server, the questionnaire underwent an online pretest at the police academy among students to assess practicality and quality so minor mistakes and errors could be corrected in advance.

The questionnaire was based on the validated and widely used NMQ by Kuorinka et al. (1987) [22], adapted in line with a modified German version of the NMQ (FB*MSB) [23], which was in the validation process at the time of the study. The FB*MSB assesses 12-month, 4-week, and 7-day prevalence across 10 body regions. In the present version, however, we focus exclusively on the 12-month and 7-day prevalence in nine regions, as the lower leg region, which is assessed separately in the FB*MSB, was excluded for reasons of time efficiency and to improve comparability. In contrast to the original NMQ by Kuorinka (1987) the FB*MSB and our questionnaire ask specifically about symptom duration and frequency (“no days,” “1–7 days,” “8–30 days,” “more than 30 days, but not every day,” and “on (almost) every day”) and if possible differentiates between the side of the body the complaints were observed. To allow for a more detailed characterization of complaint patterns, our questionnaire contains items from an assessment inventory [32] addressing physician visits as well as sick leave related to the complaints. For a better understanding of how severe complaints are, the present version included a numeric rating scale (NRS) for pain intensity (0–10 scale, with 0 being no pain at all). The NRS is a widely used and reliable instrument for measuring pain [33]. Furthermore, police-specific aspects (e.g., weapon carrying position, service-related injuries, duty-related and leisure-time physical activity) were taken into account. These modifications were intended to capture both the standard epidemiological indicators of MSDs and occupation-specific exposures in police officers. In addition, officers were provided a free text field to express assumptions about the causes of their complaints, such as body protection gear worn on duty. An expert interview with seasoned and novice officers was conducted for occupation-specific adaptation, covering topics such as body protection equipment, weapon carrying side and location, and officers’ current sporting activities. To cover these aspects ten specific questions and four general questions related to police officers’ work were included.

### Statistical evaluation

Only the fully completed questionnaires were included in the evaluation. Data formatting was conducted using Excel, encompassing plausibility checks for response behavior. The entire dataset was evaluated using SPSS Statistics 28 (IBM Deutschland GmbH, Ehningen, Germany).

First, quantitative data were tested for normal distribution (Shapiro-Wilk test). Descriptive analyses were used to describe the sample and job-specific characteristics as well as the survey results (response frequencies for individual body regions). Since no validated composite score exists for the selected set of MSD items in police officers, the Rasch model, originally developed by George Rasch in 1960 as part of psychometric theory [32], was applied to derive an overall MSD severity for each participant. The objective of the Rasch analysis was not to evaluate the performance of individual questionnaire items, but rather to obtain a unifying metric that summarizes the severity and distribution of musculoskeletal complaints. This approach provides an objective measurement that allows comparability across individuals and groups by placing results on a common scale [34]. Here, higher Rasch scores indicate more pronounced MSDs, reflecting greater severity and prevalence of musculoskeletal symptoms. It should be noted that this score served as a secondary analytical tool to aggregate various MSD locations and complaints into a single variable, while the primary focus of the study remained the assessment of specific MSD prevalence rates. For correlation calculations (between the MSD severity and age/ /body mass index (BMI)/working years), Spearman’s rank correlation coefficient was employed. For interpreting the effect size, the criteria from Evans (1996) [35] were used, categorizing values as follows: <0.2 as poor correlation, 0.2–0.4 as weak correlation, 0.4–0.6 as moderate correlation, 0.6–0.8 as strong correlation, and > 0.8 as optimal correlation. The significance level was set at 5%.

## Results

A total of 281 questionnaires were distributed to the members of the participating organizational units. Of these, 255 were returned and completed, resulting in a response rate of 90.75%.

### Prevalence of MSDs

**Fig. 1:**
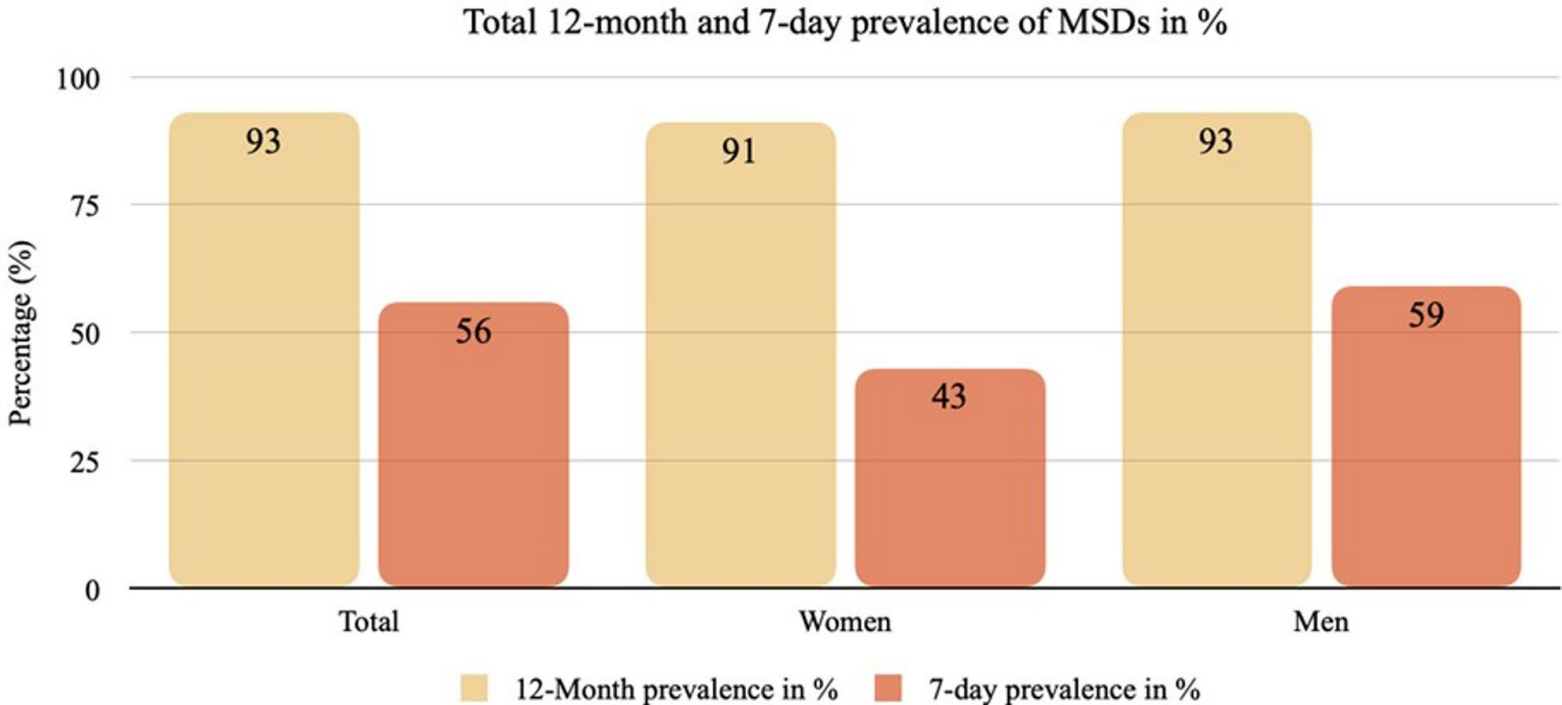
12-month and 7-day prevalence of MSDs in %

The overall 12-month prevalence of MSDs is 92.9% (*n* = 237) (Fig. 1). Only 7.1% (*n* = 18) of all respondents reported having no complaints in any body regions in the last 12 months. Multi-site pain was common: 71% of respondents reported pain in at least two body regions, and 38% in four or more. On average, respondents reported problems in three different body regions. A minority even reported having complaints in all body regions (*n* = 4; 1.6%).

The overall 7-days prevalence was 56.1%. The most affected regions largely correspond to those of the 12-month prevalence, with the difference that the first two regions are reversed. Accordingly, the most affected area is the neck/cervical spine (25.1%), followed by the lower back (20.4%), shoulder (13.7%), and thoracic spine (11.8%) (Table 1). MSDs are little more prevalent in men than in women (Fig. 1).

**Fig. 2:**
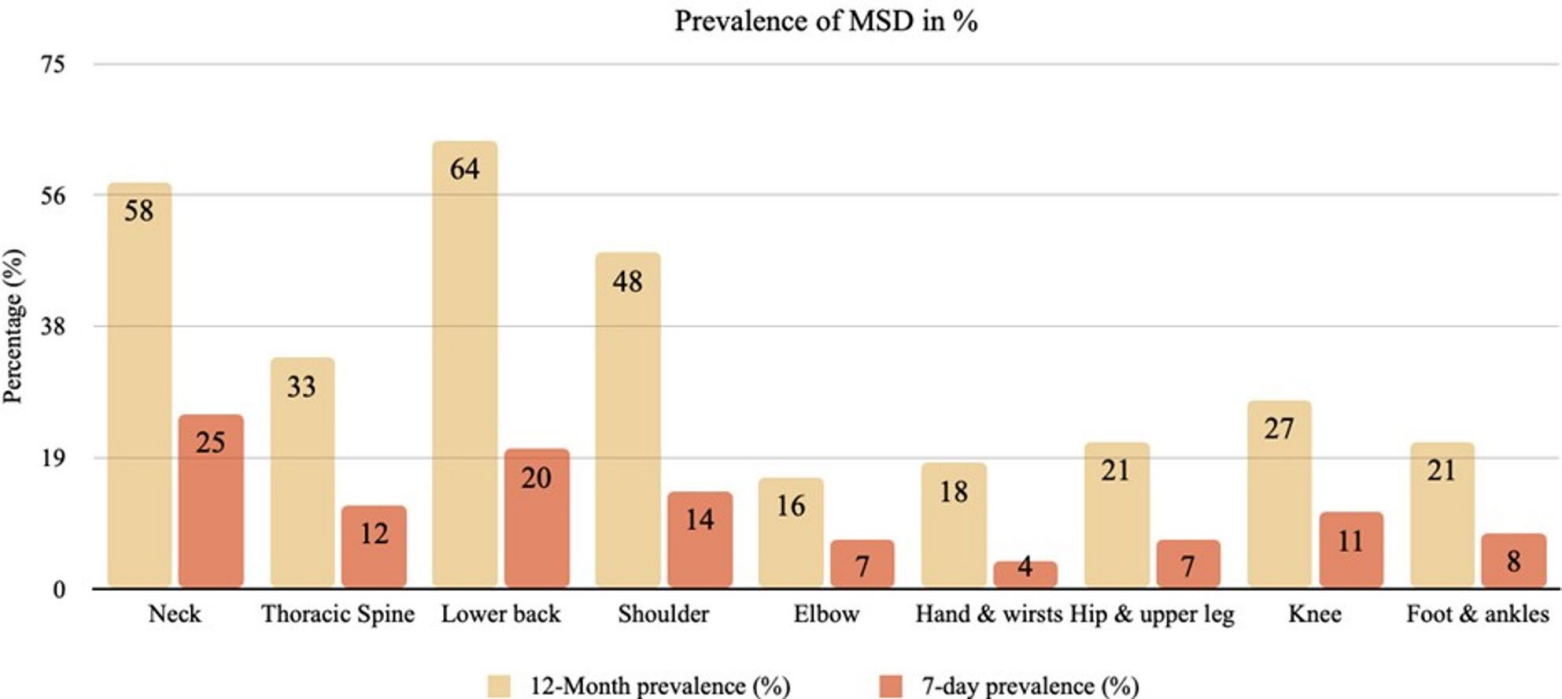
Prevalence of MSDs in the different body regions in %

Table 2 presents the prevalence and impact of musculoskeletal symptoms by body region. The highest 12-month prevalence was observed for the lower back (64.0%), neck/cervical spine (57.8%), and shoulders (48.4%), followed by the thoracic spine (32.5%) and knee (26.9%). All other regions showed 12-month prevalence rates below 25%. Figure 2 provides an overview of the distribution of MSD prevalence by body region.

Seven-day prevalence remained substantial, particularly for the neck/cervical spine (43.2%), thoracic spine (35.7%), and lower back (32.1%) among affected participants, indicating persistent symptoms. Functional limitations were frequently reported, most notably for the foot/ankle (60.4%), lower back (43.9%), knee (42.9%), and shoulder (39.2%).

The use of healthcare services varied by body region, with the highest rates of medical consultations and sick leave observed for foot/ankle, knee, and lower back complaints. Peak pain intensity was generally moderate, with the highest mean Numeric Rating Scale values reported for the lower back, knee, and foot/ankle (Table 2).

**Fig. 3:**
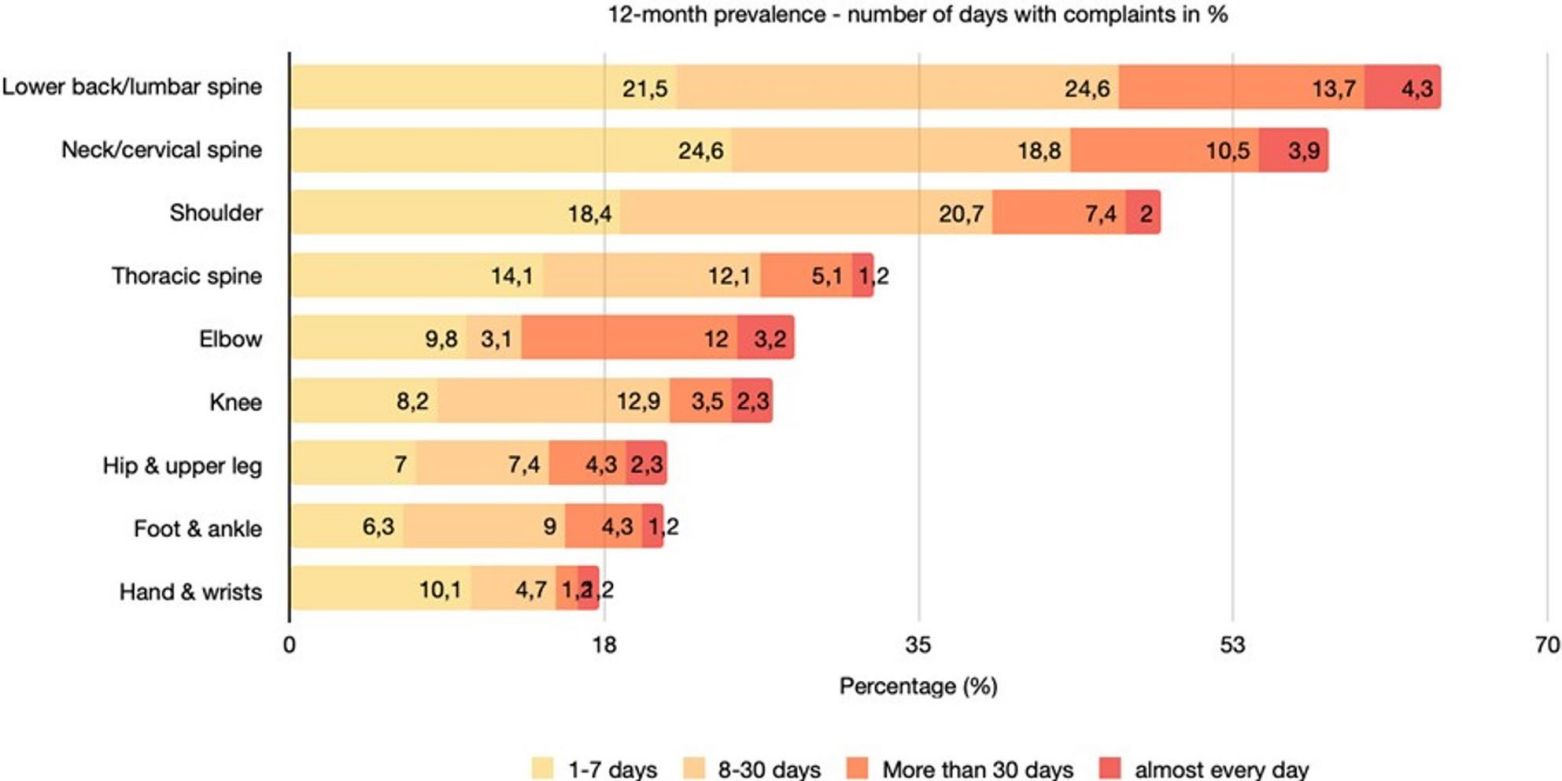
Number of days with complaints within the last 12 months in %

Figure 3 illustrates the number of days on which participants reported musculoskeletal complaints according to body region over the past 12 months. The lumbar back/lumbar spine emerged as the most frequently affected region, with 24.6% of all respondents experiencing symptoms on 8–30 days, followed by 21.5% reporting complaints on 1–7 days, and 13.7% indicating symptoms persisting for more than 30 days. The neck/cervical spine represented the second most commonly affected area, where 24.6% of participants reported symptoms on 1–7 days, and 18.8% on 8–30 days. Only a small proportion of officers experienced near-daily complaints, with the highest rate observed in the lower back region (4.3%).

**Table 2:** Results of selected items of the Nordic Questionnaire (12-month and 7-day prevalence, number of respondents with limitations in the last 12 months, medical consultation, sick leave due to complaints and average values of the NRS (ø = mean))

	12-Month Prevalence % (*n*)	Number of persons with limitations due to symptoms in the last 12 month among those affected// in total % (*n*)	7-Days Prevalence among those affected/ in total % (*n*)	Medical consultation among those affected/ in total % (*n*)	Medical certificate among those affected/ in total % (*n*)		Numeric Rating Scale peak
Neck & cervical spine	57.8 (148)	37.2/ 21.6 (55)	43.2/ 25.1 (64)	22.4/ 12.9 (33)	12.8/ 7.5 (19)		2–5 (ø 4.8)
Thoracic spine	32.5 (83)	35.7/ 11.8 (30)	35.7/ 11.8 (30)	13.1/ 4.3 (11)	13.1/ 4.3 (11)		2–5 (ø 4.7)
Lower back & lumbar spine	64.0 (164)	43.9/ 28.2 (72)	32.1/ 20.4 (52)	20.1/ 12.9 (33)	24.4/ 15.7 (40)		3–5 (ø 5.5)
Shoulder	48.4 (123)	39.2/ 19.2 (49)	28/ 13.7 (35)	16.1/ 7.8 (20)	8/ 3.9 (10)		2–7 (ø 5.2)
Elbow	16.4 (42)	45.2/ 7.5 (19)	40.5/ 6.7 (17)	33.3/ 5.5 (14)	16.7/ 2.7 (7)		2–7 (ø 5.2)
Hand & wrist	17.5 (45)	42.2/ 7.5 (19)	20.5/ 3.5 (9)	22.2/ 3.9 (10)	15.6/ 2.7 (7)		1–7 (ø 5.4)
Hip & upper leg	20.7 (53)	39.6/ 8.2 (21)	36.5/ 7.5 (19)	17/ 3.5 (9)	13.2/ 2.7 (7)		2–6 (ø 5.2)
Knee	26.9 (69)	42.9/ 11.8 (30)	40.6/ 11.0 (28)	31.9/ 8.6 (22)	29/ 7.8 (20)		3–5 (ø 5.3)
Foot & ankle	20.7 (53)	60.4/ 12.5 (32)	40.4/ 8.2 (21)	47.2/ 9.8 (25)	34/ 7.1 (18)		2–7 (ø 5.9)

### Subjectively reported causes for MSDs

**Fig. 4:**
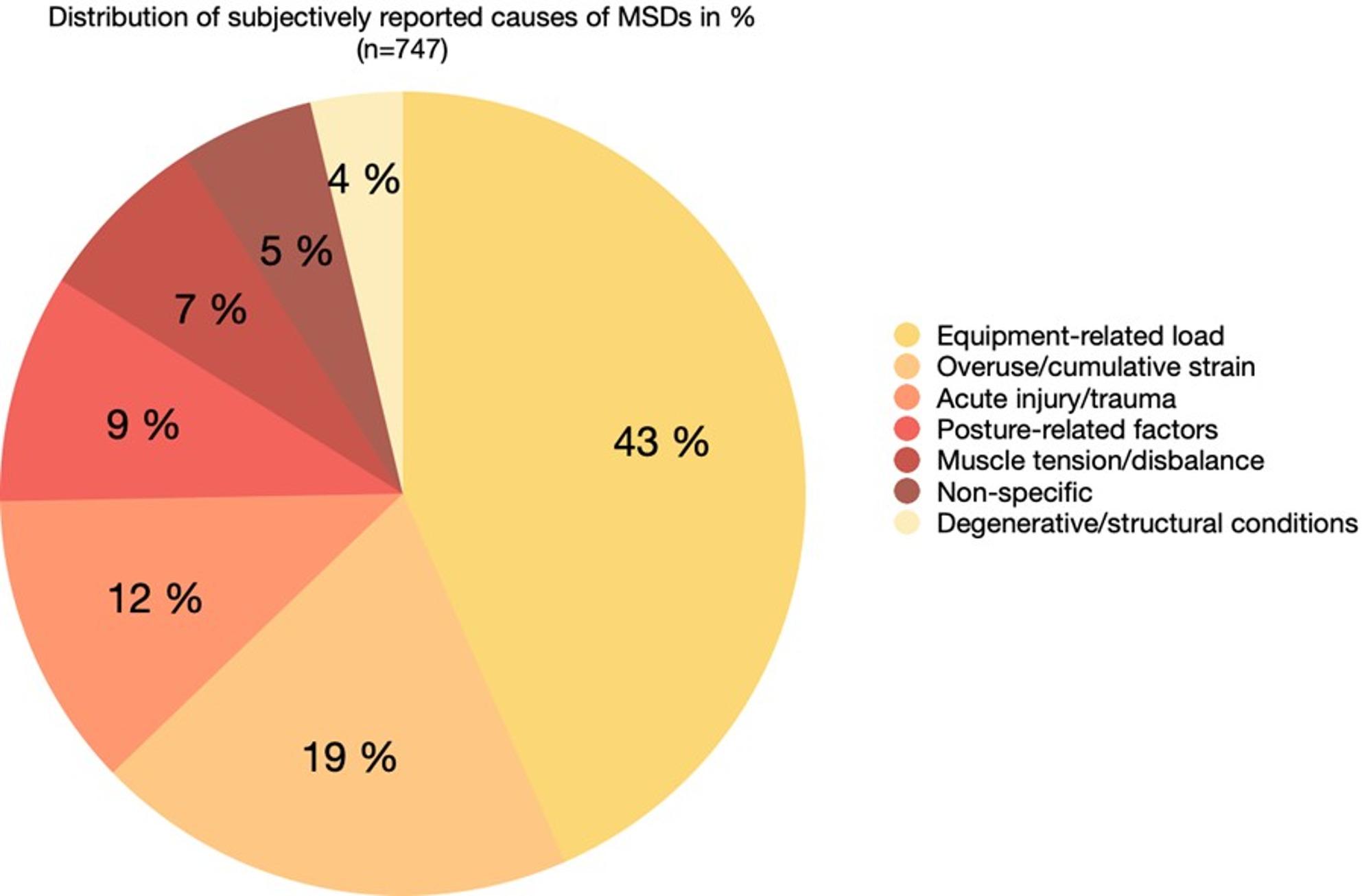
Distribution of subjectively reported causes of MSDs (*n* = 747 total causal attributions). Reported causes were grouped into higher-order thematic categories, including equipment-related load, overuse / cumulative strain, posture-related factors, acute injury or trauma, muscle tension / muscular disbalance, non-specific causes, and degenerative or structural conditions. Multiple causal attributions per individual and body region were permitted; therefore, proportions refer to the total number of reported causal attribution. Percentages may not sum to 100% due to rounding

Self-reported presumed causes of musculoskeletal complaints were grouped into higher-order thematic categories (Table 3). Hereby, free-text responses on perceived MSD causes were independently categorized by two raters. As participants frequently reported symptoms in multiple body regions, multiple causal attributions per individual and body region were permitted. Overall results are presented as frequencies and proportions of all reported causal attributions (Fig. 4), whereas region-specific findings are reported as absolute response counts (Table 3).

Across all body regions, equipment-related load (e.g., duty belts, body armor, plate carriers, helmets, prolonged wearing of protective gear) constituted the most frequently reported contributing factor (*n* = 324; 43.4% of all causal attributions). This was followed by overuse or cumulative strain (*n* = 145; 19.4%). Posture-related factors, including prolonged sitting or standing and unfavorable sleeping or head and neck positions, represented the third most common category (*n* = 69; 9.2%). Reports of previous injury or trauma accounted for 11.9% of all causal attributions (*n* = 89), whereas muscle-related complaints, such as tension or muscular disbalance, were reported less frequently (*n* = 52; 7.0%). Non-specific causes constituted a smaller proportion of attributions (*n* = 40; 5.4%), while degenerative or structural conditions (e.g., osteoarthritis, disc-related pathology) were mentioned least often (*n* = 28; 3.7%).

Clear region-specific patterns emerged across the musculoskeletal system. Complaints affecting the spine were predominantly attributed to equipment-related load, which represented the leading cause for the cervical spine, thoracic spine, and lower back, accounting for approximately 42–80% of region-specific attributions. Posture-related factors and overuse or cumulative strain constituted secondary causes across spinal regions, whereas acute injury or trauma and muscle-related complaints played only a minor role. Regarding the lower back, prolonged wear of (heavy) protective gear (especially the belt), misuse and overuse during duty (prolonged sitting), exercise (emphasizing improper weightlifting techniques), and personal factors such as herniated discs, sacroiliac joint blockages, and muscular imbalances were frequently mentioned as potential causes of issues. Additional factors were mentioned in relation to the neck, including incorrect sleeping and sitting positions.

Upper-extremity symptoms were primarily associated with equipment-related load and overuse, particularly affecting the shoulder, whereas hand, wrist, and elbow complaints were more often linked to overexertion and cumulative strain, frequently in the context of training activities, leisure-time exertion, sports-related injuries, and inflammatory conditions.

In the lower extremities, hip complaints were mainly attributed to equipment-related load, especially belt- and holster-based protective equipment, as well as restricted hip mobility and muscular imbalances, including pelvic tilt. Knee and foot complaints were predominantly associated with overexertion, prolonged standing while wearing protective equipment, acute injuries, and improper footwear.

**Table 3:** Region-specific distribution of subjectively reported causes of musculoskeletal disorders

Body region	Total attributions (*n*)	Reported causes subsumed in thematic categories (*n*; %)
Neck / cervical spine	152	equipment-related load (79; 41.6%); posture-related factors (34; 17.9%); muscle tension (19; 10.0%); overuse/cumulative strain (14; 7.4%); acute injury/trauma (6; 3.2%)
Thoracic spine	104	equipment-related load (83; 79.9%); overuse/cumulative strain (5; 4.8%); muscle tension (5; 4.8%); degenerative or structural conditions (7; 6.7%); non-specific (4; 3.8%)
Lower back / lumbar spine	174	equipment-related load (82; 47.1%); overuse/cumulative strain (33; 19.0%); posture-related factors (28; 16.1%); acute injury/trauma (15; 8.6%); muscle tension (11; 6.3%); degenerative or structural conditions (5; 2.9%);
Shoulder	96	equipment-related load (38; 36.9%); overuse/cumulative strain (27; 26.2%); acute injury/trauma (14; 13.6%); degenerative or structural conditions (6; 5.8%); non-specific (6; 5.8%); muscle tension (3; 2.9%); posture-related factors (2; 1.9%)
Hand / wrist	43	overuse/cumulative strain (20; 46.5%); acute injury/trauma (14; 32.6%); non-specific (6; 14.0%); equipment-related load (2; 4.7%); posture-related factors (1; 2.3%)
Elbow	37	overuse/cumulative strain (19; 51.4%); equipment-related load (6; 16.2%); acute injury/trauma (6; 16.2%); non-specific (6; 16.2%)
Hip / upper leg	41	muscle tension/disbalance (12; 24.0%); equipment-related load (8; 16.0%); sport/training (5; 10.0%); acute injury/trauma (5; 10.0%); posture-related factors (4; 8.0%); non-specific (4; 8.0%); overuse/cumulative strain (3; 6.0%)
Knee	54	acute injury/trauma (14; 35.2%); overuse/cumulative strain (15; 27.8%); equipment-related load (10; 18.5%); non-specific (8; 14.8%); degenerative or structural conditions (5; 9.3%); muscle tension (2; 3.7%)
Foot and ankle	46	acute injury/trauma (15; 43.5%); equipment-related load/long standing with heavy equipment (16; 34.8%); overuse/cumulative strain (4; 8.7%); non-specific (6; 13.0%); degenerative or structural conditions (5; 10.9%);

(multiple attributions per individual permitted; percentages refer to region-specific attributions)

### Correlations

Using Spearman’s rank correlation coefficient, it was examined whether the variables age, BMI, and working years have an impact on MSD severity. Significant correlations with *p* < 0.01 among all parameters and MSD severity were observed. Spearman’s correlation coefficient r and the corresponding p-values can be found in Table 4.

**Table 4:** Results of the Spearman’s rank correlation coefficient r

Correlation Parameter	Spearman correlation coefficient *r*^1^	*p*-value
Age and MSD	0.17	< 0.01
BMI and MSD	0.19	< 0.01
Working years and MSD	0.20	< 0.01

^1^ Criteria by Evans (1996): <0.2: poor correlation, 0.2–0.4: weak correlation, 0.4–0.6: moderate correlation, 0.6–0.8: strong correlation, > 0.8: optimal correlation

## Discussion

This study aimed to determine the prevalence of MSDs among police officers of a German federal state police force and to identify individual and occupational factors associated with MSD occurrence. The results demonstrate a high prevalence of MSDs (92.9%) within the past 12 months, affecting nearly all participants. Lower back (64.0%), neck/cervical spine (57.8%) and shoulders (48.4%) followed by the thoracic spine (32.5%) were the most commonly affected regions. The seven-day prevalence remained also high at 56.1%. Multi-site pain was common, suggesting that musculoskeletal strain in police work is not confined to isolated body regions but rather reflects cumulative and systemic load. Such high prevalence, despite the relatively young mean age of our sample (28 years), underscores the intensity of occupational load in German police service and suggests that cumulative strain may manifest itself early in professional life.

The observed 12-month prevalence exceeds the reported prevalence in several previous studies with police officers [13, 18–20]. For example, Canadian officers showed a 68% rate of lower back pain and Polish motorcycle police 57% overall MSD prevalence [13, 20]. In our cohort, the 64% lower-back prevalence is particularly striking, aligning with findings from Swedish police officers, where Larsen et al. [36] reported a 12-month prevalence of 61% for low back pain. This suggests that the often mentioned heavy protective equipment contributes to a sustained lumbar load. Similar mechanisms have been described by Alghamdi et al. [37] and Karakolis et al. [38], who identified prolonged static posture and the use of duty belts as key risk factors. Neck and shoulder complaints were also frequent (57.8% and 48.6%), aligning with findings from Swedish and Brazilian police samples [13, 18]. In these populations, constrained head and shoulder movement due to protective gear and the wearing of bullet-proof vests were major contributing factors. Notably, our overall 12-month prevalence of over 90% is substantially higher than the rates reported in other international studies, such as Pistolesi et al. [39], who found that over 50% of Australian police officers reported musculoskeletal complaints. This discrepancy may be attributed to the specialized nature of the participating organizational unit and the specific tactical exposures associated with their duties.

In line with previous international research [2, 12, 13, 18–21], the lower back and neck/cervical spine were the most frequently affected regions, followed by the shoulders and thoracic spine. Although pain intensity was predominantly moderate across body regions (mean scores between 4.7 and 5.9 on the numeric rating scale), a substantial proportion of officers reported functional limitations, medical consultations, and sick leave attributable to their complaints, highlighting the potential impact of MSDs on work ability and operational readiness.

When contrasted with data from the German general working population - where 12-month prevalence rates of approximately 46–49% for neck/shoulder pain and 46% for lower back pain have been reported [25] - these findings suggest that police officers experience MSD prevalences that are at least comparable (e.g. shoulder pain) and, for certain body regions (especially the lower back), elevated relative to the general working population. A comparable study involving personnel working in German emergency medical services [40] likewise demonstrated a high burden in the neck/cervical spine and lower back/lumbar spine regions; however, it showed a markedly higher 12-month prevalence in the neck/cervical spine (72.6%), whereas police officers more frequently reported low back pain.

### Occupational aspects

Several investigations confirm that duty belts, body armor, and other tactical gear markedly increase discomfort and multi-site pain, even among physically fit officers [4, 16, 41–43]. These findings are supported by the present data, as participants most frequently attributed their musculoskeletal symptoms to equipment-related load (43.4%), which accounted for the largest proportion of all reported causal attributions. Overuse or cumulative strain (19.4%) and posture-related factors (9.2%) represented further major categories, underscoring the relevance of both mechanical load and sustained static exposures in daily police work. Regarding the lower back, participants frequently referred to the prolonged wearing of heavy protective gear (especially duty belts) (47.1%), sustained sitting and standing during duty (16.1%), improper or excessive physical training, and individual factors such as disc-related pathology, sacroiliac joint dysfunction (2.9%), and muscular imbalances (6.3%) as contributing mechanisms. For cervical spine complaints, unfavorable sleeping and sitting positions (17.9%) were additionally highlighted. These subjective attributions closely reflect the objectively documented exposure profile of police work, which is characterized by extended driving times, administrative desk work, and prolonged static postures during patrol, surveillance, or crowd-control duties [44–46]. Previous studies have consistently identified prolonged sitting in police vehicles and constrained working postures as key contributors to lower back and neck pain, particularly when combined with limited opportunities for postural variation and recovery [47, 48].

In addition, the routine wearing of duty belts, ballistic vests, and auxiliary equipment—often exceeding 15–20 kg—has been shown to increase spinal compression, alter sagittal alignment, and elevate muscular activation in the trunk and shoulder girdle, thereby exacerbating mechanical load on the musculoskeletal system [49–51]. The high proportion of equipment-related attributions for spinal and shoulder complaints observed in the present study is therefore biomechanically plausible. International research demonstrates that police officers exposed to mandatory load carriage report a higher prevalence of lower back, neck, and shoulder complaints compared with officers with reduced equipment loads or alternative carrying systems [50, 52]. Studies from Sweden, Canada, and Germany further indicate that asymmetrical load distribution from duty belts and weapon carrying contributes to unilateral pain patterns and compensatory postural adaptations [15, 45, 49].

Thus, while biological factors such as age and BMI are contributing factors, ergonomic and organizational determinants - highlighted by the predominance of equipment-related, postural, and load-related attributions - appear equally critical in explaining the extraordinarily high prevalence observed. Owing to the imbalanced gender distribution in favor of male participants, gender was excluded from the analysis as it did not constitute a statistically reliable influencing factor.

It is notable that despite high physical activity levels, 95.7% of the present participants engaged in regular leisure-time sports and 78.1% in occupational training, MSD prevalence remained extremely high. This apparent paradox echoes findings from U.S. and Canadian samples, where greater physical fitness reduced but did not eliminate injury risk [24, 53]. The relationship between exercise and MSDs is complex: while physical conditioning can confer resilience, overuse or inadequate recovery may however provoke injury [15]. Future work should therefore differentiate between protective versus excessive physical training in police personnel.

### Influence of age, BMI, and years of service

The first and second hypotheses - predicting positive relationships between age, BMI, and MSDs - were verified. Although correlations were modest, the direction of effects is in agreement with extensive epidemiological evidence showing that both advancing age and higher body mass index contribute to musculoskeletal symptomatology [26–29]. This aligns with earlier findings in other policing populations. For example, a recent UK study reported a 12-month MSD prevalence of 86% among officers (mean age 40.6 years), with back, shoulder, and neck pain rates comparable to those found in our study, and similarly no strong association with age or body composition [54]. Mechanistically, age-related degenerative changes, sarcopenia, and cumulative exposure reduce the musculoskeletal system’s resilience [27]. Excessive body weight amplifies mechanical stress on joints and the spine [29], while adipose-derived cytokines (e.g., IL-6, TNF-α) induce chronic inflammation, thereby increasing tissue sensitivity and degenerative risk [26, 29]. These pathways are increasingly recognized as mediators of pain sensitization and connective-tissue deterioration [55].

Our data also verified the third hypothesis, suggesting a link between years of service and MSD severity, albeit with a weak correlation (rho = 0.20). This likely reflects cumulative exposure to occupational stressors such as load carriage, prolonged sitting, and repetitive microtrauma [9–16]. Similar associations have been reported in a large Korean police cohort, where longer tenure and irregular shift work significantly increased MSD risk [56].

Although the observed correlations reached statistical significance (*r* = 0.17–0.20), the effect sizes remain below the threshold for a weak correlation. Consequently, these associations should be interpreted with caution, as their practical relevance may be limited.

### Implications for prevention

Taken together, these findings highlight the need for preventive strategies that address both individual and organizational determinants of MSDs in policing [17, 46, 57, 58]. Early interventions targeting ergonomic optimization of equipment, load distribution, and task organization may be particularly relevant, as even weak associations with age, BMI, and years of service can translate into substantial cumulative burden over the course of a policing career [28, 59]. The fact that 96% of officers were physically active indicates that physical inactivity is not the primary issue [60, 61]; rather, training content and timing should be better aligned with occupational demands. Specific strengthening and mobility routines focusing on the back and neck regions might be more beneficial than general fitness activities [62–64]. The integration of standardized MSD assessment with police-specific contextual factors in the present study provides a valuable foundation for future longitudinal research aimed at disentangling causal pathways and informing targeted prevention efforts [17, 56, 65].

### Study limitations and future directions

Several limitations must be considered. First, the cross-sectional design precludes any conclusions regarding causal relationships between the identified risk factors and the prevalence of musculoskeletal disorders. Longitudinal research is required to confirm temporal relationships between occupational exposure and MSD development. Second, data were self-reported and may be subject to recall bias or subjective interpretation. Third, the sample was limited to one police unit in a single federal state, which restricts generalizability of the findings to the broader police force or other tactical units. Fourth, the results of this study may be subject to the ‘healthy worker effect.’ Since the data collection was conducted among active-duty officers, those on long-term sick leave -potentially due to severe musculoskeletal disorders - were not included in the sample. Consequently, the reported prevalence rates may represent an underestimation of the true burden of MSDs within this specialized organizational unit.

Fifth, no objective ergonomic or biomechanical measurements were taken. Nevertheless, the relatively large sample size (*n* = 255) and the use of a validated and modified NMQ instrument strengthen the reliability of our findings. Finally, a gender-specific analysis was not performed, as the study was primarily designed to assess overall MSD prevalence and was not a priori powered for subgroup comparisons; this limitation has been noted to avoid potential post-hoc bias and to guide future research designs.

Future research should include longitudinal monitoring and biomechanical assessments to quantify cumulative load and identify modifiable ergonomic factors. A tiered diagnostic system, such as the model proposed by Grifka et al. [66] or Holzgreve et al. [67], may offer a useful framework for occupational health programs, balancing efficiency and diagnostic accuracy. Longitudinal research is essential to track MSD progression and identify early predictors of chronic disability or career change. Comparative analyses between special forces, patrol units, and administrative officers could help clarify how task profiles influence MSD risk. Integrating wearable sensor technology may allow continuous assessment of posture and movement patterns during duty. Finally, intervention studies examining the effectiveness of targeted exercise or equipment redesign would provide practical guidance for police health management.

## Conclusion

Nearly all officers in our survey experienced musculoskeletal discomfort within the past year. Lower back and neck regions were most frequently affected, while shoulder and thoracic spine complaints were also common. Physical inactivity was not a dominant risk factor in this cohort; rather, occupational load and anthropometric characteristics appeared to play a stronger role.

Comprehensive ergonomic and organizational interventions are needed to mitigate this burden. Addressing both biomechanical and psychosocial aspects of police work is essential to maintain operational readiness and long-term health.

## Data Availability

No datasets were generated or analysed during the current study.

## Abbreviations

NMQ: Standardised Nordic questionnaires for the analysis of musculoskeletal symptoms
MSD: Musculoskeletal disorder
BMI: Body mass index
FB*MSB: Questionnaire on musculoskeletal complaints (German version)
